# Only a few positive post-intervention values in fitness measures after structured and semi-structured school-based physical activity interventions

**DOI:** 10.3389/fpsyg.2026.1844426

**Published:** 2026-08-11

**Authors:** Corrado Lupo, Gennaro Boccia, Damiano Li Volsi, Paolo De Pasquale, Alexandru Nicolae Ungureanu, Paolo Moisè, Anna Mulasso, Luca Beratto, Alberto Rainoldi, Paolo Riccardo Brustio

**Affiliations:** 1 Department of Medical Sciences, University of Turin, Turin, Italy; 2 Neuromuscular Function Research Group, School of Exercise and Sport Sciences, University of Turin, Turin, Italy; 3 Department of Clinical and Biological Sciences, University of Turin, Turin, Italy; 4 School of Exercise and Sport Sciences, University of Turin, Turin, Italy; 5 Department of Life Sciences and Systems Biology, University of Turin, Turin, Italy

**Keywords:** active children, non-active children, physical interventions, physical test, school activity

## Abstract

This study aimed to compare changes in 9–11 years old Italian children for fitness outcomes, following a structured multicomponent intervention, The Daily Mile, and usual school physical education lessons (i.e., control group). Before and after three different 6-month weekly PAIs (2 h of structured physical workouts, with a sport science expert; 4 units of The Daily Mile + 1 h of physical activity with the generalist teacher; control group, 2 h of physical activity with the generalist teacher), 263 children were anthropometrically measured (stature, body mass), and evaluated by means of physical tests regarding sprint (20 m-sprint), endurance (shuttle-run), balance (single-leg-stance), dominant upper-limb strength (handgrip), lower-limbs muscle power (standing-long-jump), and flexibility (sit-and-reach) physical fitness qualities. To distinguish active and non-active children, participants filled the Physical Activity Questionnaire for Older Children – Italian version (PAQ-C-it) at PRE. Post-intervention outcomes were analyzed using linear mixed-effects models (with PRE values, age and PRE–POST ΔBMI as covariates). For standing long jump, the structured PAI determined higher post-intervention values than the control group (estimated difference = 18.55 ± 4.6 cm, *p* = 0.001, d = 1.03) and The Daily Mile group (estimated difference = 10.89 ± 3.09 cm, *p* = 0.008, d = 0.6). For single-leg stance with eyes open, the structured PAI group achieved higher post-intervention values than The Daily Mile group (estimated difference = 25.10 ± 8.16 s, *p* = 0.016, d = 0.7). No other difference emerged between PAIs. In addition, male reported better shuttle run higher post-intervention values (*p* = 0.008, ηp^2^ = 0.03) than female. No effects for sex-intervention-physical status interactions emerged. Therefore, PAI effects are limited and outcome-specific, with only structured approach able to promote some fitness enhancements, regardless from the beginning physical status of students, thus suggesting to moderately consider the association between school PAIs and substantial fitness positive post-intervention variations.

## 1 Introduction

Regular physical activity (PA) is the key health-promoting behavior across the lifespan. According with the U.S. Department of Health and Human Services (HHS), increasing movement and reducing sedentary time provide substantial health benefits regardless of age, sex, or fitness level (US Department of Health and Human Services, 2018). Current international guidelines recommend that children and adolescents accumulate at least 60 min per day of moderate-to-vigorous physical activity, while also limiting sedentary behavior, particularly recreational screen time (Konstabel et al., 2014; National Center of Epidemiology S a. HPS e obesità, n.d.). However, a substantial proportion of young people do not meet these recommendations (Konstabel et al., 2014). In Italy, for example, only about one in four children aged 6–9 years achieves the recommended amount of daily PA, whereas a similar proportion spends at least four or more hours per day watching television (National Center of Epidemiology S a. HPS e obesità, n.d.). This scenario is quite negative especially considering that only regular engagement in PA may support the development of fundamental movement skills during childhood, enhancing motor competence and movement confidence (Fühner et al., 2021). In addition, active behaviors established during childhood and adolescence tend to persist into adulthood (Fühner et al., 2021). Accordingly, identifying feasible and effective school-based strategies to promote physical activity and improve fitness-related outcomes in children is an important public health and educational priority (Nevill et al., 2020).

Among school-based PA interventions, structured multicomponent programs may be especially relevant because they can target several components of health-related fitness simultaneously, and should be leaded by sport science expert instead of a generalist teacher. When designed according to established recommendations (US Department of Health and Human Services, 2018), these interventions may combine aerobic exercise, fundamental movement skill practice, and strength- or coordination-oriented tasks, thereby providing a broad and potentially transferable training stimulus. According to studies applied in Europe, PA interventions in schools should be regarded as a simple, non-expensive and enjoyable way to reach all the children and adolescents with adequate doses of moderate to vigorous PA, determining to benefits such as preventing obesity and cardiovascular risks among youths also by decreasing BMI, and improving metabolic parameters and physical fitness (Mura et al., 2015). Also school-based PA interventions among children and adolescents in the Middle East and Arabic speaking countries are characterized by a multi-component scenario where education components on lifestyle and diet resulted significant (Alalawi et al., 2023). Moreover, in Latin America, time spent in PA at school, and more especially the time of PA spent at moderate and vigorous intensity, resulted fundamental for promoting a successful intervention in terms of physical fitness improvements (Ribeiro et al., 2010). For this reason, it is crucial that fundamental movement skills be developed from an early age, as they contribute to numerous benefits, including reducing sedentary behavior, improved physical fitness, enhanced self-esteem and athletic performance, sustained engagement in physical activity across the lifespan, increased willingness to take risks, and stronger muscles and bones (Hands, 2012).

As an alternative to more structured programs, schools may adopt ecological or semi-structured approaches aimed at increasing daily movement opportunities. Among the latter, The Daily Mile represents a voluntary run (i.e., individually paced) over a distance of approximately one mile of distance or 15 min of time^1^ (Brustio et al., 2020). The presumed benefit of this activity in schools, which is very popular in the United Kingdom, lies primarily in its brevity and, therefore, the absence of drawbacks such as disrupting school organization and budgets (Brustio et al., 2019; Malden and Doi, 2019; Wilson et al., 2016). Furthermore, walking and/or running does not require any skill on the part of teachers or students (Wilson et al., 2016). Importantly, teachers, who play a crucial role in implementing The Daily Mile, have recognized the intervention’s potential to improve physical activity and thus prevent important health outcomes such as childhood obesity (Malden and Doi, 2019). Furthermore, previous studies conducted in the primary school context suggest that The Daily Mile may improve aerobic capacity after 3 (Brustio et al., 2019) and 8 months of participation (Chesham et al., 2018), while also contributing to favorable changes in body composition, moderate-to-vigorous PA, and sedentary behavior (Chesham et al., 2018).

Although both interventions may increase PA opportunities during school hours and positively influence children’s well-being, they rely on different intervention logics and may therefore induce distinct adaptations in children’s fitness. Structured multicomponent sessions are expected to provide a broader motor and physiological stimulus, whereas The Daily Mile may preferentially influence aerobic-related outcomes because of its predominantly repetitive walking/running format. From an applied perspective, comparing these approaches is relevant because school-based interventions should be judged not only by their efficacy, but also by their feasibility and scalability within real educational settings. Against this the above-described scenario, it remains unclear whether structured multicomponent activity and The Daily Mile produce comparable advances in fitness among Italian primary-school children, and whether these responses differ according to baseline PA status (i.e., active, non-active). Therefore, the aim of the present study was to compare changes in fitness outcomes following a structured multicomponent intervention, The Daily Mile, and usual school physical education lessons (i.e., control group) in Italian children aged 9–11 years, while also examining whether intervention responses differed according to baseline PA status and sex. We hypothesized that both intervention groups would show higher post-experimental period values than the control group.

## 2 Methods

### 2.1 Subjects

Three hundred children and relative parents/guardians gave their written informed consent after receiving detailed information about the aims and procedures of the study. Absent subjects on one of the 2 days of data collection (*n* = 37) were excluded, determining a final sample of 263 children (girls *n* = 124, 47.1%) aged 9–11 participating in this study. Information regarding the study aims, design, and procedures was first provided to teachers and parents/guardians. The same information was then explained to the children before baseline testing. All participants were recruited from four primary schools (mean *n* = 66 ± 24, range *n* = 35–90, a school) and 15 classes in total (mean *n* = 18 ± 3, range *n* = 12–22, a class) in in municipalities just outside Turin city (Northwest Italy) (Supplementary material S1, S2). All schools belonged to the same regional educational system (i.e., common teaching modules) and followed comparable curricular indications (Brustio et al., 2019), with the same weekly and daily schedule. School classes were randomly allocated to one of the three intervention conditions (structured intervention, The Daily Mile, or control) at the beginning of the study. Allocation was performed at the class level while ensuring, whenever possible, the inclusion of at least one class per intervention condition within each participating school. One exception to this allocation was required because of school organizational needs. According to the ethical standards in the Declaration of Helsinki, children, parents/guardians, and teachers provided written informed consent for the study, and before data collection, the local institutional review board approved this study (Protocol #134691).

### 2.2 Design

This was a 6-month cluster-randomized, school-based intervention study in which intact school classes were randomly allocated to one of three physical activity intervention conditions: structured intervention (6 classes, *n* = 110); The Daily Mile (6 classes, *n* = 104), or usual school physical activity lessons (control group; 3 classes, *n* = 49), respecting the inclusion of a minimum of one class within a single school per PAI (excepting for one case because of school practical needs). In fact, no information about the beginning active and non-active status has been provided to students and their teachers, making interventions equally practicable for all students of a single class. In addition, each teacher followed a single class, and university personal for physical fitness measures has no information about students’ physical status and PAI assigned. As baseline (October–November 2021), participants filled in the Italian version of the PAQ-C (PAQ-C-it) and performed the PRE-session physical tests. The 6-month intervention period then took place from October/November 2021 to March/April 2022, after which participants completed the POST assessment. Not all classes began these phases simultaneously (3 weeks between the first and last student), but all participants respected the same test and PAI volumes. In fact, the same week volume (2 h) has been maintained for the three PAIs: 2 h of physical class with a sport science expert for the structured group; 1 h of physical class plus four The Daily Mile sessions (15-min each one) with the generalist teacher for semi-structured group; and 2 h of physical class with a generalist teacher for the control group. The adherence to each PAI resulted high (<15% of individual absence to PAI days; a mean of 3.5 ± 0.2 The Daily Mile units for week), and structured PAI and The Daily Mile have been conducted by means of no progression or periodization, maintaining a quite homogeneous external load along the entire intervention period, and carrying a teaching progression from very simple physical exercitations to medium difficult one, from basically individual executions to grouped ones.

For both test sessions (PRE and POST PAI), classes were tested during school hours, between 8:30 a.m. and 12:30 p.m. Physical and anthropometric measurements were collected in randomized order in small groups of 5–6 children, depending on class size. Endurance run fitness was assessed at the end of each testing session in subgroups of 6–7 children per trial. Test sessions were conducted and supervised by the same qualified investigators (Master’s Degree in Sports Sciences), using standardized protocols and were carried out in school gymnasiums with comparable surfaces and available space in order to minimize environmental variability and ensure participant safety. During the test session, participants were verbally encouraged to perform the best trial in each test, and a not-recorded trial to familiarize with each test execution has been regularly allowed.

### 2.3 Physical activity interventions

#### 2.3.1 Structured PAI: physical activity sessions according to HHS guidelines

In accordance with HHS guidelines (US Department of Health and Human Services, 2018), each structured session has been led by a professional Master’s Degree in Sports Sciences and organized into three 20-min segments focusing on: (1) fundamental movement skills, (2) strength-based activities, and (3) aerobic activity.

The first exercise segment has been regularly introduced by basic body movements that served as the foundation for more complex motor skills, following a “simple-to-complex” instructional progression. In particular, this part has been characterized by locomotor skills such as: running and jumping; stability and balance such as static and dynamic postural control exercises (e.g., tandem stance, single-leg stands, and heel-to-toe walking); and object control such as manipulative catching and throwing to improve hand-eye coordination. Then, the strength-based activities were mainly performed to stimulate all major muscle groups (legs, hips, back, abdomen, chest, shoulders, and arms) by means of foundational patterns such as squats, hinges (deadlifts), pushes (push-ups/overhead press), and pulls (rows), executed within 8–12 repetitions (2–4 sets), at an intensity that leads to muscular fatigue, and only with body weight, resistance bands, or free weights (no gym machines). Finally, the aerobic session part consisted in performing vigorous endurance run activities through continuous, rhythmical movements using large muscle groups such as running, brisk cycling (10 + mph), jumping rope, or vigorous aerobic dancing. Sometimes, these activities have been integrated with fundamental sport-specific skills to promote physical literacy, recognized as essential for developing the confidence, competence, and motivation required for lifelong physical activity (US Department of Health and Human Services, 2018).

#### 2.3.2 Semi-structured PAI: The Daily Mile

The activity called The Daily Mile (n.d.) consisted of 15 min of outdoor walking/running performed during school hours within the school grounds. Specifically, for this subgroup, a typical physical activity week was structured with one hour of curricular physical activity in a day, plus four 15-min outings to participate in The Daily Mile activity in each of the other four weekly school days. These activities were led by the classroom teacher, under the supervision of an expert (i.e., Master’s Degree in Sports Sciences).

#### 2.3.3 Control group: curricular physical activity

For this subgroup, school physical activity remained the curricular one, led by a designated generalist teacher (i.e., teacher employed for other subjects), and not by a sport science expert (as the Italian government established only from the period after the present data collection, for the 4th and 5th primary school year).

### 2.4 Procedure

#### 2.4.1 Italian version of the Physical Activity Questionnaire for Older Children

Before starting PAIs, self-reported PA was assessed using the Italian version of the Physical Activity Questionnaire for Older Children (PAQ-C-it) (Gobbi et al., 2016). This questionnaire consisting of ten items (despite the tenth item is not considered for the final summary score) was able to collect information on participation in different types of activity, sports, and efforts during physical education classes, during lunch, after school, in the evening, and during the weekend, within the past seven, and to recognize participants as active or non-active children (information not provided to students), according to a specific threshold validated for Italian co-aged (Lupo et al., 2022).

#### 2.4.2 Anthropometric parameters

Stature and body mass were measured using a portable stadiometer (Model 214; Seca, Hamburg, Germany) with an accuracy of 0.01 m, and an electronic scale (Model 876; Seca, Hamburg, Germany), with an accuracy of 0.1 kg, respectively. Both stature and body mass were twice measured without shoes (the mean measure has been finally considered). Body Mass Index (BMI) was calculated according to the formula: body mass divided by height squared (i.e., kg/m^2^).

#### 2.4.3 Handgrip strength

Handgrip strength was measured using a digital hand dynamometer (Baseline Smedley digital hand dynamometer, model 12–0286). During the test, participants stood with their dominant arms constantly vertical and close to the body, with the not flex on the wrist joint palm. The subjects were instructed to perform a maximal voluntary contraction with the dominant hand. Grip strength was recorded in kilograms to the nearest 0.1 kg. Participants executed two trials (1-min in between) each arm, indicating to research personal the dominant one. The best score was considered for statistical analysis.

#### 2.4.4 Balance

Balance was assessed using the single-leg stance test. Children stand barefoot on their preferred leg (Condon and Cremin, 2014) with the other knee flexed, hands on the hips, for a maximum of 120 s. The test was performed under both eyes-open and eyes-closed conditions. Timing started when the participant assumed the correct position and stopped when the supporting foot moved from its initial position or when the raised foot touched the ground. Compensatory movements of the trunk or raised leg were allowed, whereas arm movements were not permitted. Time was recorded in seconds using a stopwatch (accuracy: 0.01 s). After a partial and not registered trial, a following and registered one was considered for statistical analysis.

#### 2.4.5 Low-back flexibility

Hip and low-back flexibility were assessed using the sit-and-reach test. Each child sat on the floor with knees fully extended and ankles in neutral dorsiflexion against the sit-and-reach box (Standard Flexibility Tester, Baseline, New York, NY). From this position, participants placed one hand over the other and reached forward as far as possible while keeping the knees extended (Cornbleet and Woolsey, 1996). The score corresponded to the fingertip position on the ruler, expressed in centimeters. The best result between two individual executions (with not-fixed pause in between) was considered for statistical analysis.

#### 2.4.6 Lower-limb muscle power

The standing long-jump test was performed to assess lower-limb power. Children were instructed to jump forward as far as possible from a standing start and to land on both feet while maintaining balance. In case the child put their hands on the floor at the end of his/her jump, the trial was not considered as valid, requesting another trial. Jump distance was measured to the nearest 0.01 m from the take-off line to the nearest heel mark. In case of asymmetric landing feet, the closest foot to the take-offline was considered to measure the jump result. The best score between two performed trials (with a 1-min pause in between) was considered for statistical analysis (Castro-Piñero et al., 2009).

#### 2.4.7 Sprint ability

The 20-m-linear-sprint test was considered to evaluate the children’s sprint ability. Participants started individually with both feet behind the starting line and were instructed to run the 20-m distance as fast as possible, decelerating only after crossing the finish line. Photoelectric cells (Witty – Wireless Training Timer; Microgate, Udine, Italy) was used to register the time elapsed from the start to the finish line (measuring with an accuracy of 0.01 s). The best score between two performed trials (1-min in between) was considered for statistical analysis.

#### 2.4.8 Endurance run fitness

The 20-m shuttle-run Leger test soundtrack version (CAEP Quebec Faca) was used to evaluate the endurance run fitness. The test was performed once and consisted of running in small groups (i.e., 4–6 for single trial) back and forth (i.e., in shuttles) between two lines 20-m apart, following signals played from a pre-recorded soundtrack (beep). The test started at a speed of 8.5 km^.^h^−1^ and increased by 0.5 km^.^h^−1^ every minute (Welsman and Armstrong, 2019). The end of the test occurs when the child does not reach the line in time (i.e., before the correspondent audio signal) on two consecutive occasions, thus determining the test score in correspondence of the last reached stage (i.e., distance covered, expressed in meters) (De Miguel-Etayo et al., 2014). The test was supervised by a minimum of four investigators to supervise children’s performances and establish the end of the test for each one. After a partial and not registered trial, a following and registered one was considered for statistical analysis.

### 2.5 Statistical analysis

Descriptive data are presented as estimated marginal means and 95% confidence intervals of post-intervention values, adjusted PRE, for physical fitness tests across physical activity status (active vs. non-active), PAIs, and sex. No primary outcome was prospectively specified in the study protocol. Therefore, all physical fitness outcomes were considered of equal interest and analyzed within an exploratory framework. Differences in post-intervention outcomes were analyzed using linear mixed-effects models, with PRE values of the corresponding test included as covariates. Age and PRE–POST BMI (ΔBMI) were also included as covariates to control for potential confounding effects. Physical activity status, PAIs, and sex, along with all their interactions (physical activity status × PAI × sex), were included as fixed effects. To account for the clustered allocation of the intervention, classroom, defined as the school-by-class combination, was included as a random intercept. Denominator degrees of freedom and *p*-values for fixed effects were estimated using the Satterthwaite approximation, as implemented in the lmerTest package. Because only four schools contributed to the analytical sample, school was not included in the primary model; however, a sensitivity analysis including school as a fixed effect was performed and yielded consistent results. Model assumptions were evaluated using simulation-based residual diagnostics implemented in the DHARMa package, including tests of residual uniformity, dispersion, and outliers.

Estimated marginal means and 95% confidence intervals were derived from the linear mixed-effects models. When significant main effects or interactions were detected, *post hoc* pairwise comparisons and, where appropriate, simple-effect comparisons were estimated from the estimated marginal means. Bonferroni adjustment was applied to all multiple pairwise comparisons, including those performed following significant interactions. Adjusted mean differences, 95% confidence intervals, and Bonferroni-adjusted *p*-values were reported. The Bonferroni correction controlled the family-wise error rate within each set of *post hoc* comparisons but did not account for multiplicity across the overall set of outcomes, main effects, and interaction terms examined. Effect sizes for fixed effects were estimated using partial eta squared (ηp^2^), computed from the mixed-effects models using the effectsize package and Cohen’s d with 95% confidence intervals for pairwise comparisons. Partial eta squared values were interpreted as small (0.01), medium (0.06), and large (0.14). Cohen’s d with 95% confidence intervals was calculated for pairwise comparisons using the emmeans package. Cohen’s d effect sizes were interpreted as follows: <0.2 = trivial, 0.2–0.6 = small, 0.7–1.2 = moderate, 1.3–2.0 = large, and >2.0 = very large. Statistical significance was set at *p* < 0.05. All analyses were performed using RStudio IDE Version 2026.01.0 + 392 with the packages lme4 (Bates et al., 2015), lmerTest (Kuznetsova et al., 2017), emmeans (Lenth, 2023), and effectsize (Ben-Shachar et al., 2020).

## 3 Results

No significant three-way interaction between physical activity status, PAIs, and sex emerged for any outcome. Subjects’ POST physical fitness test results, adjusted for PRE values, age, ΔBMI, and class-level clustering, are reported in Table 1.

**Table 1:** Adjusted post-intervention estimated marginal means (95% confidence intervals) according to physical activity intervention (C = Control, S = Structured PAI, D = The Daily Mile).

Outcome	C	S	D	p PAI	ηp^2^ PAI
Handgrip dominant (kg)	18.84 (13.57 to 24.11)	16.89 (13.20 to 20.58)	19.66 (15.98 to 23.34)	0.463	0.10
Handgrip non-dominant (kg)	16.98 (11.78 to 22.19)	16.21 (12.57 to 19.85)	18.40 (14.77 to 22.03)	0.609	0.06
Sit-and-reach (cm)	20.89 (18.55 to 23.23)	23.89 (22.64 to 25.13)	22.18 (20.97 to 23.39)	0.024	0.27
Standing long jump (cm)	118.66 (110.98 to 126.34)	137.20 (132.37 to 142.04)	126.31 (121.96 to 130.67)	<0.001	0.09
Single-leg stance eyes open (s)	77.14 (56.62 to 97.67)	93.30 (81.29 to 105.31)	68.21 (56.36 to 80.05)	0.006	0.40
Single-leg stance eyes closed (s)	18.29 (5.98 to 30.60)	21.84 (14.50 to 29.18)	17.67 (10.42 to 24.92)	0.615	0.05
20 m sprint (s)	4.16 (4.05 to 4.28)	4.11 (4.04 to 4.18)	4.15 (4.08 to 4.22)	0.552	0.06
Shuttle run (m)	649.71 (533.54 to 765.88)	728.59 (652.40 to 804.77)	706.52 (625.26 to 787.78)	0.447	0.09

Values are adjusted for PRE score, age, ΔBMI, physical activity status, sex, and class-level clustering, Physical Activity Intervention (PAI).

**Figure 1:**
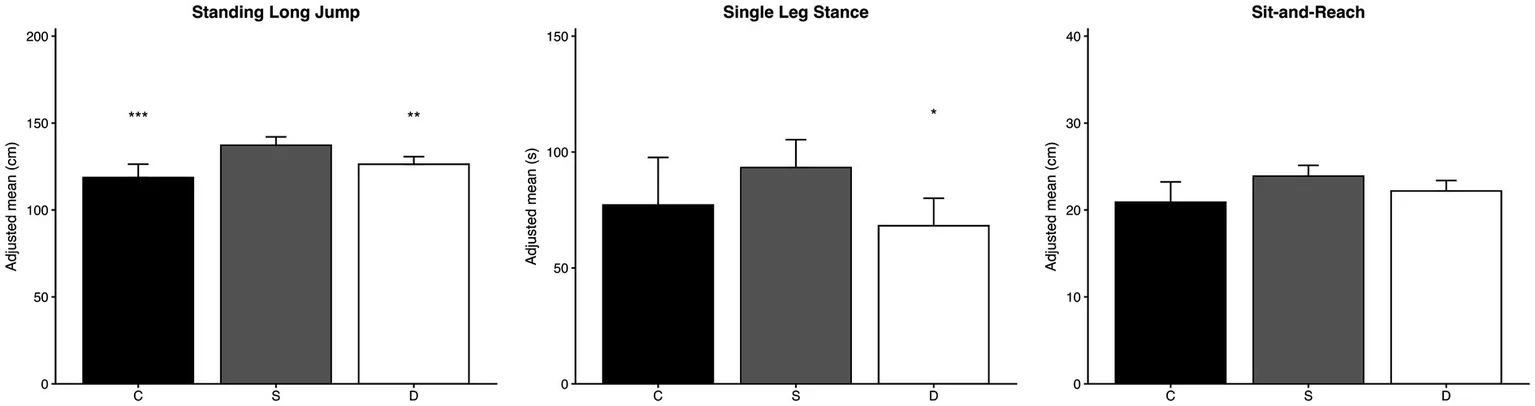
Adjusted post-intervention estimated marginal means for standing long jump, single leg stance, and sit-and-reach tests (* *p* ≤ 0.05, ** *p* ≤ 0.01 with respect to structured intervention).

PAI effects emerged for standing long jump (*p* < 0.001, ηp^2^ = 0.1), single-leg stance with eyes open (*p* = 0.006, ηp^2^ = 0.4), and sit-and-reach (*p* = 0.02, ηp^2^ = 0.3). No significant PAI effects emerged for dominant handgrip, non-dominant handgrip, single-leg stance with eyes closed, 20 m sprint, or shuttle run. *Post hoc* comparisons showed that, for standing long jump, the structured PAI group achieved higher adjusted post-intervention values than the control group (estimated difference = 18.55 ± 4.6 cm, 95% CI: 7.06 to 30.04, *p* = 0.001, d = 1.03, 95% CI: 0.48 to 1.58) and The Daily Mile group (estimated difference = 10.89 ± 3.09 cm, 95% CI: 2.69 to 19.10, *p* = 0.008, d = 0.6, 95% CI: 0.23 to 0.97). No significant difference emerged between the control group and The Daily Mile group. For single-leg stance with eyes open, the structured PAI group achieved higher adjusted post-intervention values than The Daily Mile group (estimated difference = 25.10 ± 8.16 s, 95% CI: 3.99 to 46.20, *p* = 0.016, d = 0.7, 95% CI: 0.21 to 1.13), whereas the other pairwise comparisons were not significant. For sit-and-reach, although the overall PAI effect was significant, no Bonferroni-adjusted pairwise comparison reached statistical significance (Figure 1).

**Figure 2:**
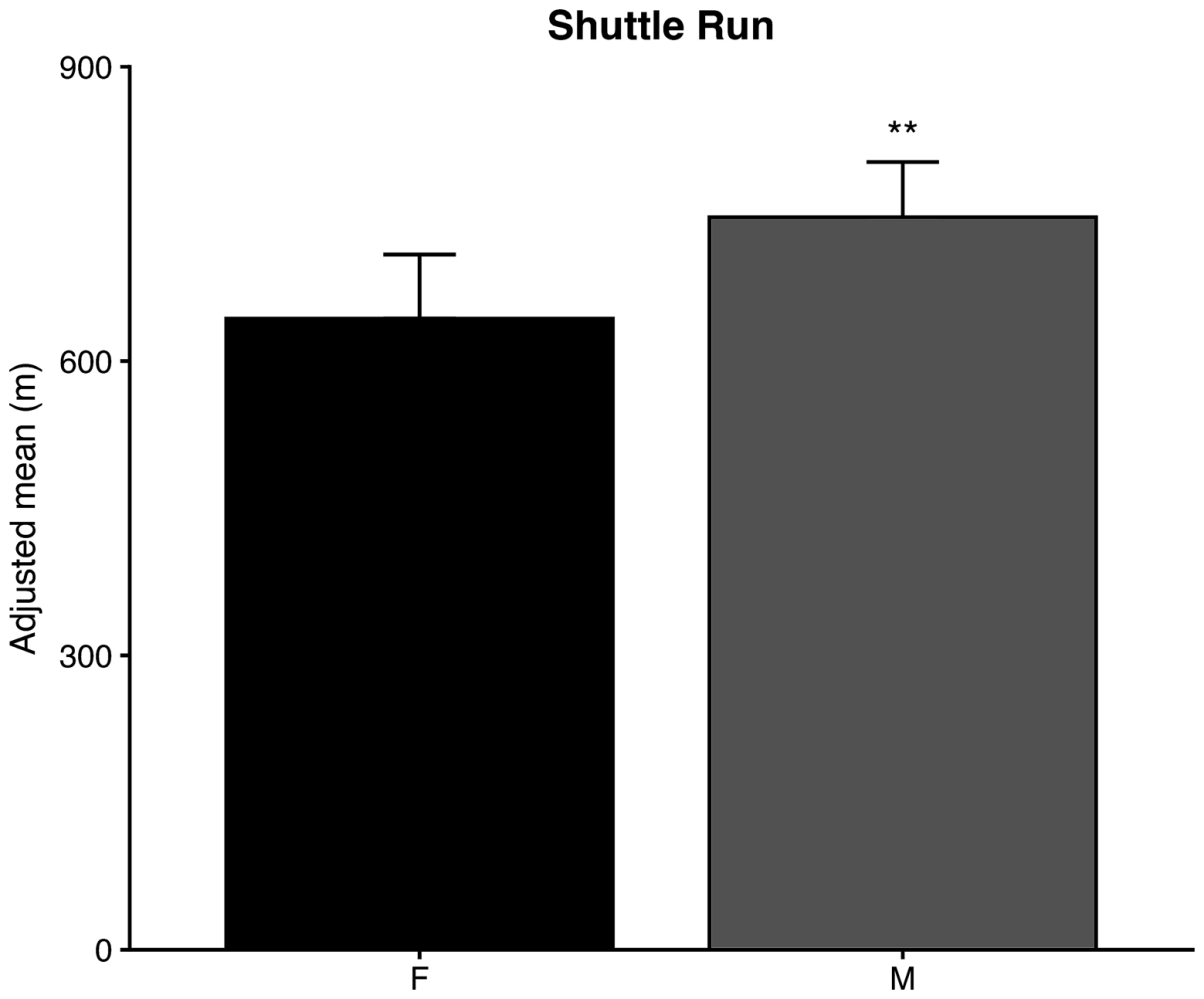
Adjusted sex estimated marginal means for shuttle-run test (** *p* ≤ 0.01 with respect to female values).

Regarding physical activity status, no significant main effect emerged for any outcome. A significant physical activity status × sex interaction emerged only for single-leg stance with eyes closed (*p* = 0.035, ηp^2^ = 0.02). Simple-effect analyses showed no significant difference between active and non-active participants within females (*p* = 0.12) or males (*p* = 0.18). Among active participants, females showed higher adjusted post-intervention values than males (estimated difference = 13.09 ± 6.60 s, *p* = 0.048, d = 0.6, 95% CI: 0.00 to 1.21), whereas no sex difference emerged among non-active participants (*p* = 0.49). For sex comparisons, a significant effect emerged only for shuttle run (*p* = 0.002, ηp^2^ = 0.04), where male participants reported higher adjusted post-intervention values than female participants (estimated difference = 103 ± 33 m, 95% CI 38.2 to 168, *p* = 0.002, d = 0.5, 95% CI: 0.18 to 0.86; Figure 2). No significant sex × PAI × physical activity status interaction emerged for any outcome.

## 4 Discussion

The present longitudinal study aimed at verifying if a school structured and a semi-structured PAI are able to determine higher post-intervention values in fitness performance in 9–11 years old Italian children. In particular, fitness performance higher post-intervention values would have been expected especially by non-active subjects, who were characterized by a lower PRE physical practice level. In particular, the inclusion of a structured school PAI led by a sport science expert, even with systematic interventions in terms of aerobic and resistance-training stimuli, or the regularity of PAI promoted by The Daily Mile might have been expected as favorable with respect to the curricular PAI, led by a designated generalist teacher, principally educated for other subjects, and thus not methodologically oriented to the students’ physical fitness progression.

Nevertheless, the main finding of the present study was that higher post-intervention values in physical fitness were limited and outcome specific, strengthening the findings of a previous cross-sectional study highlighting a missing association between practiced activity and fitness level (Lupo et al., 2023). In fact, no interaction between physical activity status, PAIs, and sex emerged for any outcome, and PAI effects specifically emerged for standing long jump, single-leg stance with eyes open, and sit-and-reach regardless of active or non-active status. However, among the examined outcomes, the clearest post-hoc effects emerged only for lower-limb muscle power, with the structured intervention producing higher post-intervention values than both The Daily Mile and the control condition. In addition, balance (i.e., single-leg-stance test executed with eyes open) reported benefit from structured PAI (despite a little ceiling effect with 15% of participants reaching the maximum of 120 s emerged only in this case) with respect to The Daily Mile one (7% at 120 s). However, no three-way interaction (PAI x sex x physical status) emerged, despite a physical activity status × sex interaction was instead observed for single-leg stance with eyes closed. As a consequence, this pattern suggests that school-based interventions may not produce uniform adaptations across all fitness domains. In addition, the general sex effect on shuttle running, where male participants better improved their endurance performance than female counterparts, strengthens the probability of a higher impact from individual grown than from specific stimuli, despite this phenomenon are quite controversial, mainly reporting similarities in this physical capability, age, and sex comparison (Vaccari et al., 2021; Baquet et al., 2003).

However, from a general point of view, the sporadic fitness performance effects following PAIs could be also considered with the absence of effects for BMI, which resulted quite in line with the 50th percentiles reported for male and female co-aged Italian children (Cacciari et al., 2006) already at the PRE step, with no difference between PAI subgroups and physical status categories, as well as no alterations at POST. Therefore, even if this is not systematically demonstrated, it could be marginally considered that fitness performance higher post-intervention values could be limited by the absence of a corresponding BMI enhancement, which can be considered as not necessary both in active and non-active participants in this circumstance. Therefore, in this case, fitness higher post-intervention values could be probably better promoted in non-active subjects only by means of a wider intervention, which is able to involve both school and extra school daily life PAIs (Fernandez-Lazaro and Fernández-Lázaro, 2023).

The results of the present study are partially consistent with a previous and similar study considering co-aged Italian children, where fitness performance has been evaluated before and after two school structured PAIs with “specialized” personal and a control group characterized by physical activities leaded by generalist teachers. In fact, BMI remained within the normal range throughout the intervention period, strengthening the hypothesis that lower-limb power test (swinging counter movement jump) was actually influenced by the two structured PAIs, differently from the control group (Lucertini et al., 2013). Similarly, in the present study, the clearest intervention-related effect emerged for lower-limb power, with the structured intervention showing a significant advantage in standing long-jump performance. However, compared with our results, Lucertini et al. (2013) reported a pattern of fitness-related improvements also including flexibility (trunk forward flexion), upper limb strength (despite only for pinch strength test and not for handgrip), and endurance (Leger’s shuttle run).

In general, this study seems to reinforce the findings of a previous cross-sectional analysis (Lupo et al., 2022) for which PA level and fitness performance are not solidly associated, despite other works demonstrated the opposite scenario for children of 8–9 years old, with positively relationships between PA level and explosive upper-limb strength, sprint performance, and flexibility in boys, and flexibility in girls (Sacchetti et al., 2012). In addition, the wide presence of normal children’s BMI in our study at PRE, and the minor portion of overweight and underweight subjects, could have limited improvements on fitness performance after PAIs (Rauner et al., 2013). Finally, considering data from other studies on Italian children’s fitness, we can note how our non-active PRE results were quite higher, especially for sit-and-reach test (Sacchetti et al., 2012; Varalda et al., 2026; Weston et al., 2019), and handgrip (Weston et al., 2019), or similar, like for 20-m sprint (Sacchetti et al., 2012; Varalda et al., 2026; Weston et al., 2019), thus tending to consider the proposed PAIs (including control groups) as able only to maintain or weakly improve fitness performances. In fact, the only exception can be found for standing-long-jump which report a lower level at PRE and quite similar in S subgroup at POST with respect to co-aged children (Sacchetti et al., 2012; Varalda et al., 2026; Weston et al., 2019; Galvani et al., 2024). Differently, for single-leg-stance, results are quite similar to previous study for PRE (Bae et al., 2015), highlighting similar advancements at POST, with a significant PAI effect for the eyes-open condition and a physical activity status × sex interaction for the eyes-closed condition. Instead, for shuttle run test, even though higher post-intervention values were reported regardless of PAI effects, male increases resulted higher than those of sex counterparts, also reporting lower level at PRE and quite similar (Weston et al., 2019) or even higher (Söğüt et al., 2019) at POST with respect to previous studies.

However, some limitations exist in this study. Firstly, the PAQ-C-it filling in could have determined some inaccuracies in terms of children’s comprehension, even though research personal dedicated care and standardizing procedures in explain each part of the questionnaire. In addition, the active and non-active discrimination has been considered according to the Italian children specific PAQ-C-it cut-off (Lupo et al., 2022) only at PRE, thus not quantifying eventual changes during the PAI period in terms of extra-school physical and sport activity attendance, and biological maturation. In fact, despite the participation of children in extra-school physical activities are generally decided at the beginning of the scholar and sportive season (in September), therefore not in the present experimental period, no data has been recorded to monitor eventual news in this way. In addition, different individual involvement experienced by participants of each single PAI and heterogenous occurrences of participants in sample subgroups (e.g., from the minimum of 3 female active C PAI participants to the maximum of 36 female active S PAI ones), could have altered effects, thus missing a solid trend. Consequently, eventual experimental replications could improve the solidity of results by monitoring this latter aspect, only selecting individual cases confirmed as active or non-active at both PRE and POST sessions. Because several physical fitness outcomes and interaction terms were evaluated, the possibility of inflated type I error across the overall set of analyses cannot be excluded. Therefore, findings, particularly those close to the significance threshold, should be interpreted with appropriate caution. Lastly, further studies would include a larger sample for each category analyzed to have a consistent occurrence of subjects in all subgroups.

In conclusion, this study on Italian children aged 9–11 years old had been able to demonstrate that school PAIs, regardless of their structured or semi-structured application, are not always and absolutely able to provide higher post-intervention values on fitness performance, both in active and non-active subgroups. As a consequence, future studies could investigate if a wider physical or sport intervention, engaging extra-scholar activities, could substantially determine substantial improvements in fitness level for children with these characteristics or with different circumstances in terms of individual BMI, socio-cultural, or geographic school environment.

## Data Availability

The raw data supporting the conclusions of this article will be made available by the authors, without undue reservation.

## Footnotes

1
http://thedailymile.co.uk/
