# Supplementary material for: Only a few positive post-intervention values in fitness measures after structured and semi-structured school-based physical activity interventions
Source: Front Psychol. 2026 Aug 11;17:1844426. doi: 10.3389/fpsyg.2026.1844426 (PMC13503234; doi:10.3389/fpsyg.2026.1844426)
Supplement: Supplementary file 1 [file Table_1.DOCX]

Supplementary table 1. Descriptive values (means ± standard deviations) of all physical fitness tests at the PRE PAI session, in relation to PAI type (control, C; structured, S; The Daily Mile, D), participants status (Active, Non-Active), and sex (male, M, female, F).

| C | | | | |
| --- | --- | --- | --- | --- |
|  | Active | | Non-Active | |
|  | M | F | M | F |
| n | 8 | 3 | 24 | 14 |
| age | 9.09 ± 0.31 | 8.89 ± 0.32 | 9.1 ± 0.42 | 9.13 ± 0.46 |
| BMI | 18.33 ± 2.93 | 17.81 ± 5.42 | 19.51 ± 3.97 | 17.67 ± 2.22 |
| PAQ-C | 3.16 ± 0.35 | 3.17 ± 0.42 | 2.34 ± 0.33 | 2.15 ± 0.41 |
| Handgrip dominant (kg) | 18.44 ± 6.43 | 9.70 ± 2.4 | 16.15 ± 3.98 | 13.16 ± 1.65 |
| Handgrip non-dominant (kg) | 16.96 ± 4.37 | 11.77 ± 1.39 | 14.48 ± 4.28 | 12.62 ± 1.90 |
| Sit-and-reach (cm) | 14.19 ± 5.81 | 24 ± 3.97 | 20.71 ± 7.62 | 21.07 ± 9.53 |
| Standing long jump (cm) | 120.13 ± 19.93 | 122.67 ± 30.09 | 120.42 ± 21.52 | 105.5 ± 13.11 |
| Single-leg stance eyes open (s) | 48.88 ± 39.1 | 25.67 ± 22.5 | 30.65 ± 30.09 | 35.46 ± 32.25 |
| Single-leg stance eyes closed (s) | 7.63 ± 5.07 | 6.67 ± 6.35 | 6.25 ± 6.28 | 10 ± 8.01 |
| 20 m sprint (s) | 4.24 ± 0.34 | 4.67 ± 0.54 | 4.48 ± 0.38 | 4.49 ± 0.33 |
| Shuttle run (m) | 592.5 ± 359.35 | 606.67 ± 382.8 | 470 ± 233.46 | 495.71 ± 244.47 |
|  |  |  |  |  |
| S | | | | |
|  | Active | | Non-active | |
|  | M | F | M | F |
| n | 28 | 36 | 24 | 22 |
| age | 9.68 ± 0.42 | 9.64 ± 0.45 | 9.69 ± 0.3 | 9.62 ± 0.45 |
| BMI | 18.97 ± 2.98 | 17.8 ± 2.41 | 18.78 ± 3.08 | 18.14 ± 3.48 |
| PAQ-C | 3.33 ± 0.42 | 3.26 ± 0.44 | 2.35 ± 0.36 | 2.32 ± 0.31 |
| Handgrip dominant (kg) | 16.62 ± 2.75 | 15.94 ± 3.6 | 15.43 ± 4.77 | 14.76 ± 3.38 |
| Handgrip non-dominant (kg) | 15.32 ± 3.1 | 15.03 ± 2.95 | 15.23 ± 4.01 | 14.33 ± 4.09 |
| Sit-and-reach (cm) | 21.68 ± 5.31 | 27.4 ± 7.03 | 19.94 ± 7.39 | 23.32 ± 7.47 |
| Standing long jump (cm) | 122.3 ± 18.97 | 117.26 ± 23.58 | 115.44 ± 21.15 | 105.98 ± 15.31 |
| Single-leg stance eyes open (s) | 43.29 ± 39.49 | 50.46 ± 41.69 | 38.46 ± 31.5 | 58.95 ± 40.84 |
| Single-leg stance eyes closed (s) | 7.25 ± 7.28 | 11.4 ± 12.01 | 8.17 ± 6.39 | 10.41 ± 9.08 |
| 20 m sprint (s) | 4.25 ± 0.4 | 4.28 ± 0.36 | 4.25 ± 0.28 | 4.48 ± 0.44 |
| Shuttle run (m) | 605 ± 322.75 | 531.43 ± 224.51 | 513.33 ± 239.69 | 467.27 ± 227.24 |
| D | | | | |
|  | Active | | Non-active | |
|  | M | F | M | F |
| n | 28 | 22 | 27 | 27 |
| age | 9.45 ± 0.76 | 9.34 ± 0.52 | 9.36 ± 0.6 | 9.29 ± 0.48 |
| BMI | 18.25 ± 2.9 | 18 ± 3.26 | 18.19 ± 2.59 | 17.68 ± 2.67 |
| PAQ-C | 3.42 ± 0.52 | 3.48 ± 0.44 | 2.23 ± 0.42 | 2.36 ± 0.24 |
| Handgrip dominant (kg) | 17.14 ± 7.44 | 14.5 ± 2.82 | 18.15 ± 5.98 | 14.96 ± 3.46 |
| Handgrip non-dominant (kg) | 16.33 ± 6.93 | 13.31 ± 3.39 | 16.58 ± 5.99 | 13.86 ± 3.44 |
| Sit-and-reach (cm) | 20.39 ± 8.68 | 24.86 ± 6.4 | 19.9 ± 6.36 | 23.33 ± 6.84 |
| Standing long jump (cm) | 122.82 ± 19.56 | 117.95 ± 14.25 | 114.63 ± 19.42 | 116.07 ± 18.35 |
| Single-leg stance eyes open (s) | 39.64 ± 32.19 | 46.41 ± 28.84 | 31 ± 34.81 | 46.04 ± 40.67 |
| Single-leg stance eyes closed (s) | 4.46 ± 3.08 | 7.68 ± 5.68 | 6.22 ± 4.64 | 8.89 ± 8.14 |
| 20 m sprint (s) | 4.17 ± 0.33 | 4.28 ± 0.35 | 4.28 ± 0.37 | 4.41 ± 0.4 |
| Shuttle run (m) | 644.55 ± 321.66 | 520 ± 235.13 | 568.7 ± 348.02 | 376.84 ± 159.2 |
