# Supplementary material for: Only a few positive post-intervention values in fitness measures after structured and semi-structured school-based physical activity interventions
Source: Front Psychol. 2026 Aug 11;17:1844426. doi: 10.3389/fpsyg.2026.1844426 (PMC13503234; doi:10.3389/fpsyg.2026.1844426)
Supplement: Supplementary file 2 [file Table_2.DOCX]

Supplementary table 2. Descriptive values (means±standard deviations) of all physical fitness tests at the POST PAI session, in relation to PAI type (control, C; structured, S; The Daily Mile, D), participants status (Active, Non-Active), and sex (male, M, female, F).

| C | | | | |
| --- | --- | --- | --- | --- |
|  | Active | | Non-Active | |
|  | M | F | M | F |
| n | 8 | 3 | 24 | 14 |
| BMI | 19.07 ± 2.96 | 18.19 ± 4.95 | 19.63 ± 4.19 | 17.88 ± 3.27 |
| Handgrip dominant (kg) | 17.5 ± 3.54 | 17.23 ± 5.08 | 16.93 ± 3.95 | 15.32 ± 2.15 |
| Handgrip non-dominant (kg) | 15.2 ± 2.69 | 16.73 ± 4.8 | 15.32 ± 3.62 | 13.86 ± 1.63 |
| Sit-and-reach (cm) | 15.69 ± 6.37 | 20.5 ± 4.95 | 20 ± 8.28 | 20.96 ± 10.12 |
| Standing long jump (cm) | 123.12 ± 30.96 | 117.33 ± 22.5 | 123.33 ± 23.97 | 106.21 ± 34.26 |
| Single-leg stance eyes open (s) | 73.50 ± 35.82 | 84 ± 50.91 | 61.12 ± 46.95 | 84.57 ± 37.38 |
| Single-leg stance eyes closed (s) | 9.13 ± 12.02 | 32 ± 41.01 | 12.33 ± 11 | 14.64 ± 11.24 |
| 20 m sprint (s) | 4.1 ± 0.42 | 4.39 ± 0.59 | 4.22 ± 0.34 | 4.35 ± 0.39 |
| Shuttle run (m) | 802.5 ± 446.12 | 606.67 ± 386.95 | 650 ± 355.53 | 564.29 ± 317.99 |
|  |  |  |  |  |
| S | | | | |
|  | Active | | Non-active | |
|  | M | F | M | F |
| n | 28 | 36 | 24 | 22 |
| BMI | 19.12 ± 3.03 | 18.16 ± 2.48 | 18.95 ± 3.11 | 18.22 ± 3.92 |
| Handgrip dominant (kg) | 19.4 ± 4.43 | 18.5 ± 3.77 | 20.16 ± 5.1 | 16.05 ± 4.44 |
| Handgrip non-dominant (kg) | 18.18 ± 2.99 | 17.25 ± 4.49 | 20.43 ± 3.77 | 16.12 ± 5.19 |
| Sit-and-reach (cm) | 22.57 ± 6.66 | 29.14 ± 7.26 | 19.83 ± 7.37 | 25.52 ± 7.13 |
| Standing long jump (cm) | 139.93 ± 28.91 | 140.70 ± 27.19 | 136.65 ± 28.98 | 130.44 ± 19.31 |
| Single-leg stance eyes open (s) | 101.96 ± 36.7 | 98.67 ± 32.62 | 78.67 ± 45.02 | 102.05 ± 36.56 |
| Single-leg stance eyes closed (s) | 21.18 ± 19.32 | 30.94 ± 34.67 | 22.79 ± 33.06 | 17.64 ± 13.34 |
| 20 m sprint (s) | 4.1 ± 0.42 | 4.08 ± 0.37 | 4.12 ± 0.38 | 4.23 ± 0.4 |
| Shuttle run (m) | 823.57 ± 420.2 | 705.56 ± 291.89 | 791.67 ± 303.83 | 572.38 ± 274.19 |
| D | | | | |
|  | Active | | Non-active | |
|  | M | F | M | F |
| n | 28 | 22 | 27 | 27 |
| BMI | 18.49 ± 3.2 | 18.61 ± 2.96 | 18.61 ± 3.25 | 18.03 ± 2.89 |
| Handgrip dominant (kg) | 22.25 ± 9.95 | 19.4 ± 7.25 | 18.77 ± 6.16 | 16.45 ± 3.95 |
| Handgrip non-dominant (kg) | 20.18 ± 10.22 | 18.2 ± 6.72 | 17.91 ± 5.81 | 15.46 ± 3.96 |
| Sit-and-reach (cm) | 21.28 ± 8.86 | 25.84 ± 7.53 | 19.31 ± 7.18 | 22.15 ± 7.97 |
| Standing long jump (cm) | 137.74 ± 16.72 | 127.09 ± 25.39 | 119.63 ± 24.59 | 121.04 ± 20.01 |
| Single-leg stance eyes open (s) | 68.14 ± 43.12 | 82.9 ± 44.01 | 62.2 ± 44.46 | 62.00 ± 46.24 |
| Single-leg stance eyes closed (s) | 10.29 ± 12.27 | 21.76 ± 24.64 | 18.32 ± 29.86 | 18.58 ± 13.2 |
| 20 m sprint (s) | 3.97 ± 0.24 | 4.12 ± 0.35 | 4.2 ± 0.42 | 4.27 ± 0.35 |
| Shuttle run (m) | 803.70 ± 375.56 | 693.64 ± 245.52 | 728.89 ± 401.55 | 537.5 ± 195.28 |
